# Research on the Relationship between Water Diversion and Water Quality of Xuanwu Lake, China

**DOI:** 10.3390/ijerph15061262

**Published:** 2018-06-14

**Authors:** Weiwei Song, Qing Xu, Xingqian Fu, Peng Zhang, Yong Pang, Dahao Song

**Affiliations:** 1 College of Hydrology and Water Resources, Hohai University, Nanjing 210098, China; songweiwei0515@163.com; 2 College of Environment, Hohai University, Nanjing 210098, China; zhap2014@163.com; 3 School of Hydraulic, Energy and Power Engineering, Yangzhou University, Yangzhou 225009, China; 4 Kewen College, Jiangsu Normal University, Xuzhou 221116, China; 18852196181@163.com (X.F.); 18852196517@163.com (D.S.); 5 Key Laboratory of Integrated Regulation and Resources Development on Shallow Lakes, Ministry of Education, Hohai University, Nanjing 210098, China

**Keywords:** water diversion, degradation coefficient, model simulation, optimal plan, water quality improvement

## Abstract

Water diversion is often used to improve water quality to reach the standard of China in the short term. However, this large amount of water diversion can not only improve the water quality, but also lead to a decline in the water quality (total phosphorus, total nitrogen) of Xuanwu Lake. Through theoretical analysis, the relationship between water quality and water diversion is established. We also found that the multiplication of the pollutant degradation coefficient (*K*) and the water residence time (*T*) is a constant (*N*), K⋅T=N. The water quality changed better at first, with the increase of inflow discharge, and then became worse, and the optimal water quality inflow discharge is 180,000 m^3^/day. By constructing two-dimensional hydrodynamic and water quality models, the optimal diversion water plan is calculated. Through model calculations, it can be seen that reducing the inflow discharge makes the water residence time longer (15.3 days changed to 23.8 days). Thereby, increasing the degradation of pollutants, and thus improving water quality. Compared with other wind directions, the southwest wind makes the water quality of Xuanwu Lake the most uniform. The concentration of water quality first became smaller and then became larger, as the wind speed increased, and eventually became constant. Implementing these results for water quality improvement in small and medium lakes will significantly reduce the cost of water diversion.

## 1. Introduction

Water transferred from one place to another has been used worldwide for irrigation, flood control, water supply, power generation, and so on. In many countries, transfer of a large quantity of low-nutrient water to a eutrophic lake is considered one approach for lake restoration. The theory behind this mechanism is that adding large amounts of low-nutrient water would reduce net nutrient loading and increase the flushing rate in a lake, and consequently lower the steady state of nutrient concentration and the likelihood of algae biomass [1]. Inter-basin water transfer engineering, conveying water artificially from a present surplus to deficit catchment/river for redistributing much needed water supplies, changing living conditions, and the ecological environment, has been successfully used in many places in the world [2]. Over 160 large-scale inter-basin water transfer projects have been built-in 24 countries especially in Canada, the United States, the former Soviet Union, and India, etc. [3]. In China, water transfer projects have further increased in recent years. The famous Great Canal with a total length of 1794 km from Beijing to Hangzhou city is the longest and first manual-canal in the world mainly for navigation. Also, the South-to-North Water Diversion Project is another well-known water transfer project in China [4]. It is regarded as a strategic and ambitious approach to resolve water shortage problems in the north with three transfer routes delivering water from a different reach of the Yangtze River to the north of China facing water shortage [5]. These historical inter-basin water transfer projects have traditionally helped navigation, irrigation, water supply, hydropower, and flood control, etc. However, with the development and utilization of water resources, water quality problems of inter-basin water transfer have also begun to appear and are becoming more and more serious.

Lakes are the connecting points of interactions between the atmosphere, biosphere, soil circles, and terrestrial hydrosphere. They play an irreplaceable role in regulating regional climate, recording regional environmental changes, maintaining regional ecosystem balance, and diversifying derivatives. The rapid urbanization, industrialization, and highly accelerated economic development in China has resulted in excessive water consumption and degradation of water resources. Serious water pollution reduces the use of water features, increasing pollution-induced water shortage city, further aggravating the water shortage. Some urban lakes (like Taihu lake, Chaohu lake, Dianchi lake) and landscape lake water ecosystem are impaired due to point and non-point sources, which originate from a wide variety of human activities [6]. Urban lakes, characterized as small water bodies located in cities, act as important recreation and flood regulation sites. They are usually slow-flow, shallow water bodies with municipal pipe networks around them, leading to deterioration of water quality [7]. Urban lakes are likely to be particularly susceptible to the effects of water management and human activities [8]. Owing to the lack of municipal sewage pipeline construction in China, the overflow of sewage pipes around lakes can cause contamination with pollutants and lead to water quality degradation [9]. With intensive economic development, the consequences of this kind of pollution are increasingly serious. External pollution sources, internal sources (nutrient release from sediment), and hydrodynamic conditions (local circulation) are all critical factors resulting in water quality degradation [10]. Therefore, the implementation of water quality improvement projects in heavily polluted urban lakes is essential for water management. Inter-basin water transfer from other clean water bodies is an effective and widely used method to relieve water quality deterioration [11,12].

After pollutants enter the water body and migrate with the water stream, they are affected by factors such as hydraulics, hydrology, and the physics and chemistry in the process of migration, causing the transport, mixing, decomposition, dilution, and degradation of pollutants. The purpose of establishing a water environment mathematical model is to identify the relationship between these mutual constraints and to provide a basis for the planning, control, and management of the water environment. With the advancement of computing, monitoring and communication technologies, the river and lake water environment simulation and prediction technologies have also been continuously improved. At present, there are many models for simulating the hydrodynamic processes of rivers and lakes, such as QUAL2K (River and Stream Water Quality Model), MIKE, WASP (Water Quality Analysis Simulation Program), and EFDC (Environmental Fluid Dynamic Code) [13]. Water quality models, indispensable tools of supporting water quality predictions, have been widely applied in environmental management in recent years [14,15,16,17,18].

The paper takes Xuanwu Lake in Nanjing as an example. Xuanwu Lake is accompanied by typical developed cities. High-intensity human interference has resulted in deterioration of lake water quality and loss of water functions. At the same time, Nanjing is located in the lower reaches of the Yangtze River and its water quality is poor. A considerable amount of water diversion from the Yangtze River, not only does not improve the water quality of Xuanwu Lake, but also causes the water quality to deteriorate. In the absence of good water, the water quality can be also improved by increasing the water residence time in Xuanwu Lake and increasing the degradation of pollutants. By collecting relevant water quantity and water quality data (2014~2017), the optimal water quality inflow discharge of Xuanwu Lake was analyzed. We further predicted the impact of different wind direction and wind speed on the water quality under the conditions of optimal water diversion. The paper will provide examples for the management of small and medium-sized urban lakes, and also provide a scientific basis for the management of the water environment in Xuanwu Lake.

## 2. Study Area and Methods

### 2.1. Study Area

**Figure 1:**
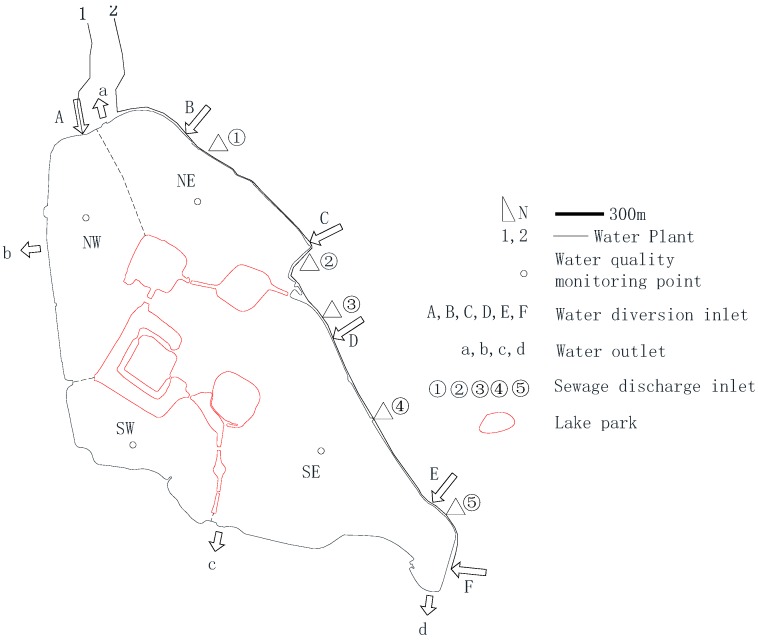
Study area.

Xuanwu Lake is a typical urban shallow lake with an area of 5.5 km^2^, of which the water area is 3.7 km^2^. When the surface elevation is 10 m, the average water depth is 1.14 m, and the storage capacity is 4,290,000 m^3^. The highest water level is 11.15 m, and the minimum water level is 9.8 m, perennial water level 9.8 m to 10.2 m. The Xuanwu Lake area is a warm and humid subtropical climate. The average annual temperature is 15 °C to 16 °C. Generally, the highest temperature is from July to August, averaging about 28 °C. The average annual precipitation is about 1000 mm. The annual wind speed is 8 m/s, and the prevailing wind direction is southeast wind and northwest wind. Since 2000, Xuanwu Lake has implemented water diversion, and the water supply from Water Plant has been 50,000 m^3^/day. In 2003, the l water supply was 180,000 m^3^/day, and in 2004, it was 280,000 m^3^/day. The water sources for diversion are all taken from the Yangtze River, and are sent to Xuanwu Lake through dedicated pipelines after sedimentation. Xuanwu Lake supplies water through two pipelines (1, 2) of waterworks and is divided into six inlets (A, B, C, D, E, F). Four outlets (a, b, c, d) drain the city rivers, and five drainage ditches (① ② ③ ④ ⑤) discharge sewage to Xuanwu Lake. The Xuanwu Lake is divided into the northeast district (NE), northwest district(NW), southeast district (SE) and southwest district (SW). Under normal circumstances, the No. 1 water plant pipeline has a flow rate of 80,000 m^3^/day. It only supplies water to the A inlet. The No. 2 water plant pipeline has a flow of 200,000 m^3^/day and supplies water to the BCDEF inlets. There is a water diversion capacity of 70,000 m^3^/day upstream of the ③ drainage ditch. Xuanwu Lake has maintained a water diversion of 280,000 m^3^/day in normal conditions. Under special circumstances, the ③ drainage ditch is used to transfer water. The total water diversion capacity of Xuanwu Lake is 350,000 m^3^/day, as shown in Figure 1. Depending to the results of water quality assessment, the main water pollution factors in Xuanwu Lake are total phosphorus (TP) and total nitrogen (TN). Annual water quality cannot reach Class IV (TP ≤ 0.1 mg/L, TN ≤ 1.5 mg/L) under every day water diversion.

### 2.2. Study Methods

#### 2.2.1. Hydrodynamic Two-Dimensional Model

The Cartesian coordinate system of two-dimensional hydrodynamic governing equations is the continuity equations and momentum equations for the integral of the three-dimensional Renault Navier-Stokes equations [19,20] of the incompressible fluid along the direction of water depth, which can be expressed as follows:

Continuity equation:

(1) $$\frac{\begin{array}{c} \partial h \end{array}}{\partial t}+\frac{\partial h\bar{u}}{\partial x}+\frac{\partial h\bar{v}}{\partial y}=hQ$$

Momentum equation:

(2) $$\frac{\partial h\bar{u}}{\partial t}+\frac{\partial h{\bar{u}}^{2}}{\partial x}+\frac{\partial h\bar{vu}}{\partial y}=fh\bar{v}-gh\frac{\partial \eta }{\partial x}-\frac{h}{\rho_{0}}\frac{\partial P_{a}}{\partial x}-\frac{gh^{2}}{2\rho_{0}}\frac{\partial \rho }{\partial x}+\frac{\tau_{sx}}{\rho_{0}}-\frac{\tau_{bx}}{\rho_{0}}-\frac{1}{\rho_{0}}\left(\frac{\partial S_{xx}}{\partial x}+\frac{\partial S_{xy}}{\partial y}\right)+\frac{\partial }{\partial x}\left(hT_{xx}\right)+\frac{\partial }{\partial y}\left(hT_{xy}\right)+hu_{s}Q$$

(3) $$\frac{\partial h\bar{v}}{\partial t}+\frac{\partial h{\bar{v}}^{2}}{\partial y}+\frac{\partial h\bar{vu}}{\partial x}=-fh\bar{u}-gh\frac{\partial \eta }{\partial y}-\frac{h}{\rho_{0}}\frac{\partial P_{a}}{\partial y}-\frac{gh^{2}}{2\rho_{0}}\frac{\partial \rho }{\partial y}+\frac{\tau_{sy}}{\rho_{0}}-\frac{\tau_{by}}{\rho_{0}}-\frac{1}{\rho_{0}}\left(\frac{\partial S_{yx}}{\partial x}+\frac{\partial S_{yy}}{\partial y}\right)+\frac{\partial }{\partial x}\left(hT_{xy}\right)+\frac{\partial }{\partial y}\left(hT_{yy}\right)+hv_{s}Q$$

where *t* represents time; *x*, *y* represent Cartesian coordinates; *h* represents total water depth; *η* represents water level; ρ represents water density; $\bar{u}$ and $\bar{v}$ represent average water depth; *f* = 2*Ωsinφ* denotes the Coriolis factor (*Ω* is the angular velocity of the Earth’s rotation, *φ* is the geographical latitude); $S_{xx}$, $S_{xy}$ and $S_{yy}$ are the radiation stress tensors; *P_a_* is the atmospheric pressure; *Q* is the point source emissions; g is the gravitational acceleration;

(4) $$h\bar{u}=\int_{-d}^{\eta }udz,h\bar{v}=\int_{-d}^{\eta }vdz,$$

where: $\rho_{0}$ represents the relative density of water; $\left(u_{s},v_{s}\right)$ represents the rate at which the outside world is released into the waterbody.

Transverse stress, *T_ij_*, includes viscous resistance, turbulent frictional resistance, and differential advection frictional resistance, which can be calculated using the eddy viscosity equation of the mean vertical velocity:

(5) $$T_{xx}=2A\frac{\partial \bar{u}}{\partial x},T_{xy}=A\left(\frac{\partial \bar{u}}{\partial y}+\frac{\partial \bar{v}}{\partial x}\right),T_{yy}=2A\frac{\partial \bar{v}}{\partial x}.$$

#### 2.2.2. Water Quality Two-Dimensional Model

##### Basic Equations of Water Quality Model

The water quality equation is built on the mass balance equation. The three-dimensional water quality transport equation contains a lot of uncertain parameters. Under the existing conditions, the verification of the model is difficult. Considering factors, such as data and model calculation workload, the average vertical two-dimensional water quality model is adopted [21,22]. The two-dimensional water quality transport equation is:

(6) $$\frac{\partial C_{i}}{\partial t}+U\frac{\partial C_{i}}{\partial x}+V\frac{\partial C_{i}}{\partial y}=\frac{\partial }{\partial x}\left(E_{x}\frac{\partial C_{i}}{\partial x}\right)+\frac{\partial }{\partial y}\left(E_{y}\frac{\partial C_{i}}{\partial y}\right)+K_{i}C_{i}+S_{i},$$

where: *C_i_* is the pollutant concentration; *u*, *v* are the flow velocity components in the *x* and *y* directions, respectively; *E_x_* and *E_y_* are the diffusion coefficients in the *x* and *y* directions, respectively; *K_i_* is the pollutant degradation coefficient; *S_i_* is the pollutant sediment release item.

In order to introduce a quantitative relationship between sediment resuspension flux and hydrodynamic conditions in the model and reflect the change of resuspension flux of each pollutant in the sediment with the flow velocity, sediment resuspension flux is calculated using the relationship obtained from sediment resuspension experiments when establishing the mathematical model [21,22], which mainly reflects the handling of the source sink term *Si*, as follows:

(7) $$S_{i}=\frac{\alpha_{i}}{H},$$

where: $\alpha_{i}$ is the sediment resuspension flux (g/(m^2^∙d)), $\alpha_{i}=\zeta_{i}\cdot \beta_{i}\exp\left(\xi_{i}\cdot P\right)$; *H* represents water depth (m); *β_i_* is the proportion of sediment pollutants in SS (%); P represents co-velocity (cm/s), $P=\sqrt{u^{2}+v^{2}}$; $\zeta_{i}$, $\xi_{i}$ are the sediment resuspension parameters.

### 2.3. Model Setup and Calibration

#### 2.3.1. Model Setup

**Figure 2:**
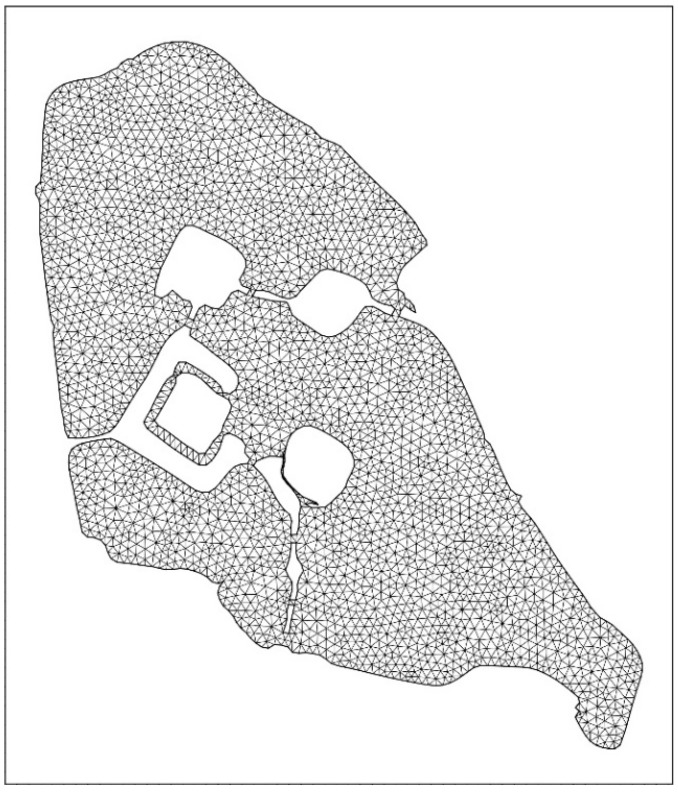
Xuanwu Lake model grid.

**Figure 3:**
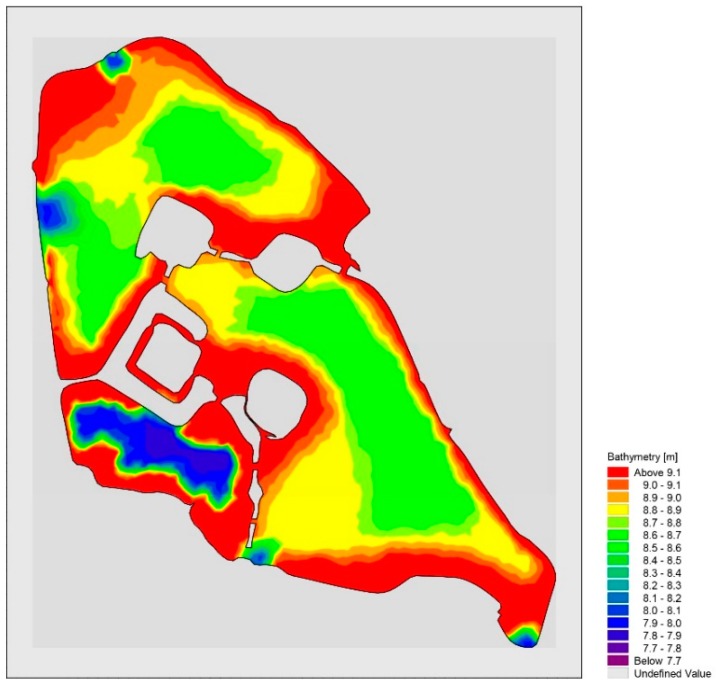
Xuanwu Lake model terrain elevation.

In the model calculation, the Xuanwu lake is divided into a three-quadrangle mixed grid with a grid spacing of about 20~30 m [23,24]. Suppose the initial time the lake is stationary, there is no disturbance, the time step *t* = 1 day [19,20]. The Xuanwu Lake model grid and the elevation elevations are illustrated in Figure 2 and Figure 3. The overall elevation of Xuanwu Lake varies from 0.8 to 2.2 m, among which the southwestern lake is the deepest, 2.2 m [25,26]. Depending on the actual topography of wading area, the geology and geographical location of the model, the Manning coefficient is 38 m^1/3^/s and the eddy parameter is 0.28 [27,28].

#### 2.3.2. Boundary Conditions

**Figure 4:**
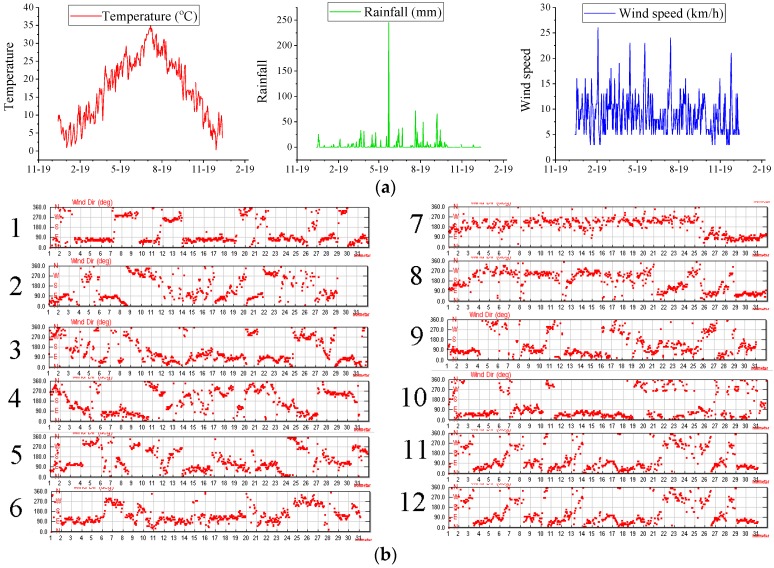

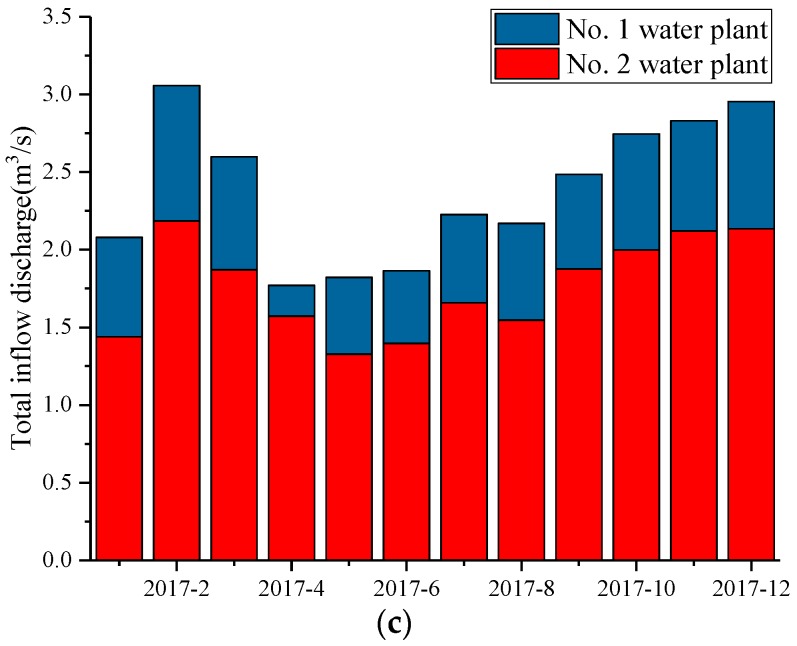
(**a**) Daily temperature, rainfall and wind speed of Xuanwu Lake in 2017; (**b**) Daily wind direction of Xuanwu Lake in 2017; (**c**) Monthly total inflow discharge of Xuanwu Lake in 2017.

Hydrodynamic: The initial water level was set to 10 m, the temperature was 10 °C, and the flow rate was set to 0 at the beginning [29,30,31]. Outlet a, b, c, d water level were set to 10 m. The temperature, rainfall, and wind speed for the whole year of 2017 are shown in Figure 4a, and the wind direction is shown in Figure 4b. The average total monthly inflow discharge is shown in Figure 4c. Meteorological data from the Internet: https://www.wunderground.com/history/airport/ZSNJ/2000/1/23/DailyHistory.html?req_city=Nanjing&req_state=&req_statename=China&reqdb.zip=&reqdb.magic=&reqdb.wmo=.

**Figure 5:**
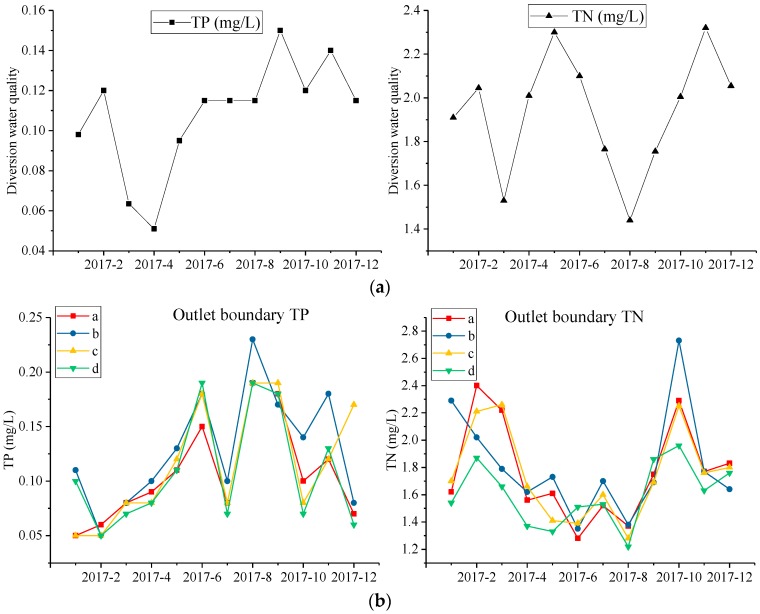
(**a**) Diversion water quality of Xuanwu Lake in 2017; (**b**) Outlet boundary water quality of Xuanwu Lake in 2017.

Water quality: The water quality (TP, TN) of inflow water is shown in Figure 5a, and the boundary of the outlet water quality (TP, TN) is shown in Figure 5b. Water quality data was supplied by the Nanjing Environmental Protection Bureau.

#### 2.3.3. Model Calibration

**Figure 6:**
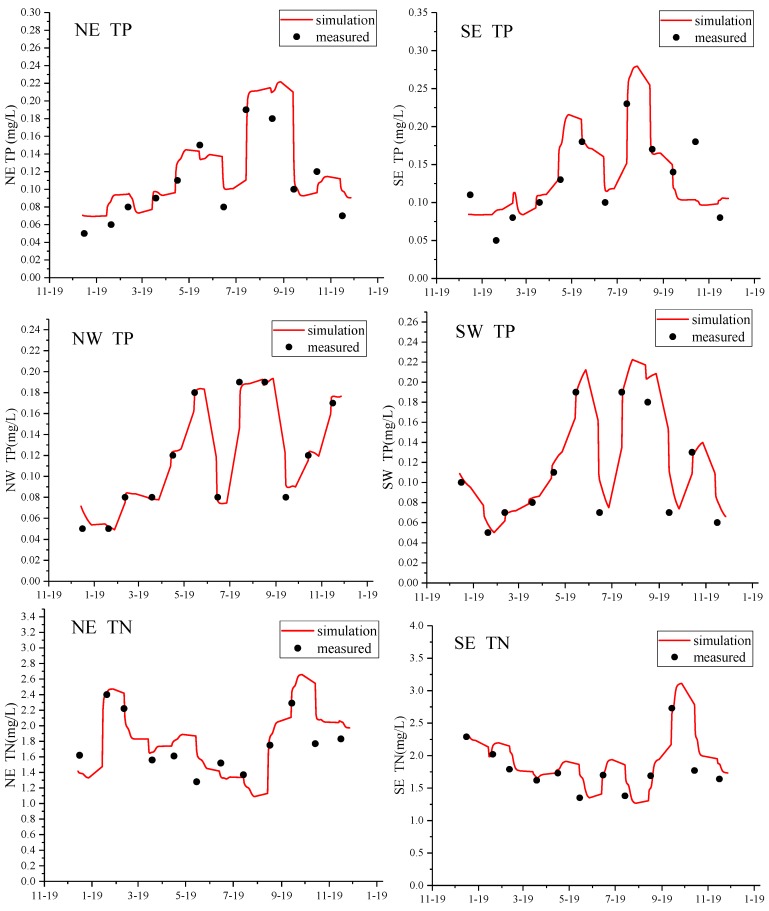

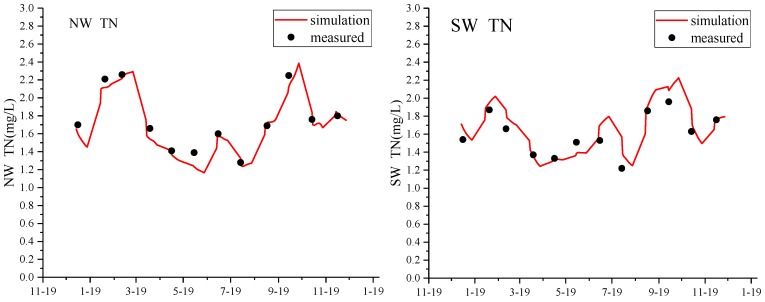
Model (water quality) validation of Xuanwu Lake in 2017.

This paper used the trial and error method to verify the model. Depending on the water quality model calculation [32,33,34], the degradation coefficient (K) of Xuanwu Lake TP was 5.92 × 10^−7^ s^−1^~2.26 × 10^−6^ s^−1^, and the TN degradation coefficient (K) was 8.55 × 10^−7^ s^−1^~1.41 × 10^−6^ s^−1^. The water quality (TP, TN) of the NE lake, SE lake, NW lake, and SW lake of Xuanwu Lake was respectively verified. The results are shown in Figure 6. It can be seen from the Table 1 that the error of calculated and measured values of water quality in the whole lake in different months were all under 20% [35,36,37]. This shows that the model can be utilized to calculate programs.

## 3. Results and Discussion

### 3.1. Analysis of the Relationship between Water Diversion and Quality of Xuanwu Lake

#### 3.1.1. Measured Data Analysis

**Figure 7:**
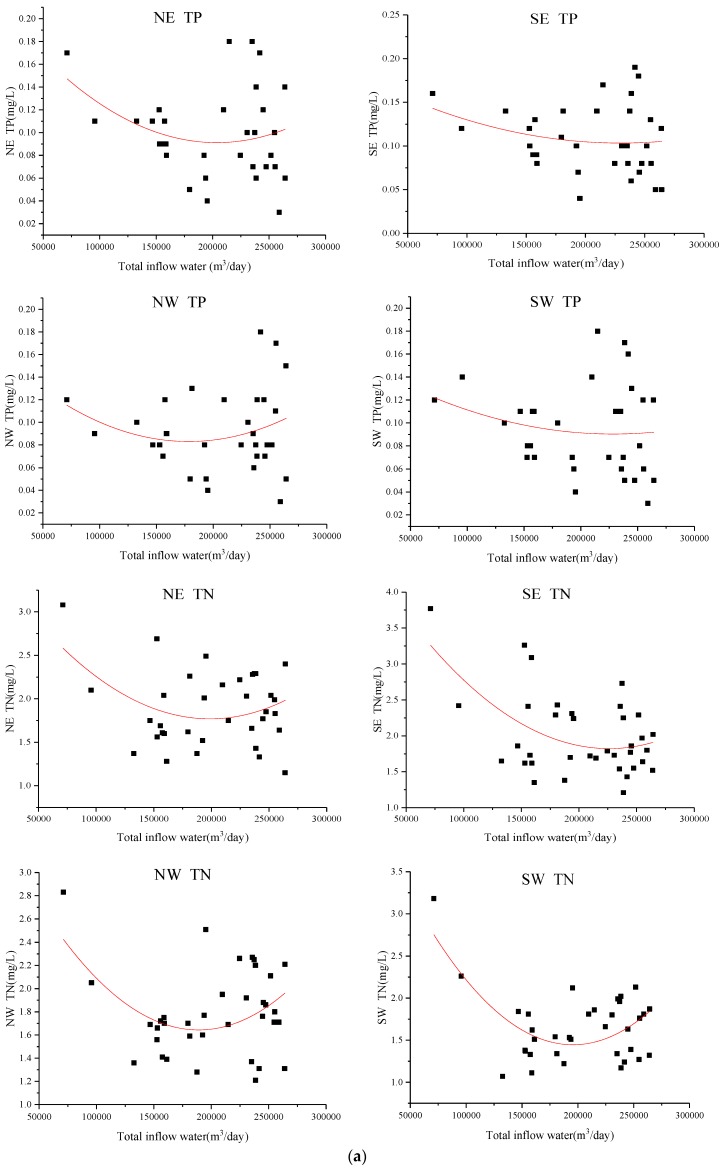

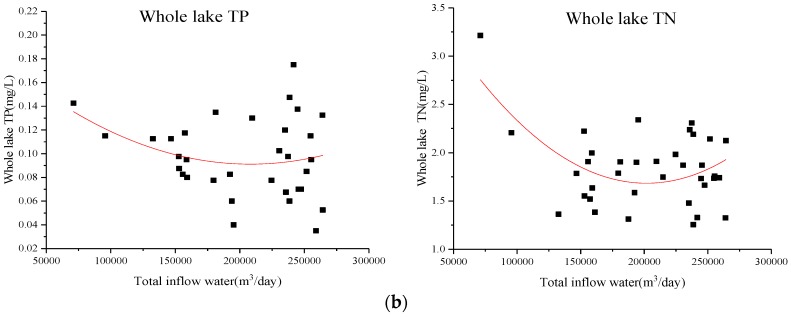
(**a**) Correlation between water diversion and quality of different lake districts measured data 2014~2017. We fit each scatter plot to a quadratic function. The equation is: *C* = Intercept + B1*Q + B2*Q^2^, *C* is the water quality concentration, *Q* is the inflow discharge, and Intercept, B1, and B2 are all constant parameters. The Intercept for each curve (left to right, top to bottom) is: 0.2235, 0.1868, 0.1718, 0.1569, 3.7432, 4.8821, 3.6543, 4.6763. The B1 for each curve (left to right, top to bottom) is: −1.299 × 10^−6^, −7.264 × 10^−7^, −9.958 × 10^−7^, −5.834 × 10^−7^, −1.986 × 10^−5^, −2.705 × 10^−5^, −2.128 × 10^−5^,−3.303 × 10^−5^. The B2 for each curve (left to right, top to bottom) is: 3.19 × 10^−12^, 1.5812 × 10^−12^, 2.7912 × 10^−12^, 1.277 × 10^−12^, 4.995 × 10^−11^, 5.979 × 10^−11^, 5.628 × 10^−11^, 8.442 × 10^−11^. The coefficient of determination, R-squared, for each curve (left to right, top to bottom) is: 0.0795, 0.0486, 0.0518, 0.0319, 0.130, 0.275, 0.178, 0.370; (**b**) Correlation between water diversion and quality of whole lake measured data 2014~2017. We fit each scatter plot to a quadratic function. The equation is: *C* = Intercept + B1*Q + B2*Q^2^, *C* is the water quality concentration, *Q* is the inflow discharge, and Intercept, B1, and B2 are all constant parameters. The Intercept for each curve (left to right) is: 0.1933, 4.2413. The B1 for each curve (left to right) is: −9.8109 × 10^−7^, −2.535 × 10^−5^. The B2 for each curve (left to right) is: 2.3564 × 10^−12^, 6.2811 × 10^−11^. The coefficient of determination, R-squared, for each curve (left to right) is: 0.0714, 0.2707.

The data of water diversion and quality (TP, TN) of Xuanwu Lake from 2014 to 2017 were analyzed, and the correlation between water diversion and water quality in each district was constructed. The curve of water quality and daily water inflow discharge (m^3^/day) in each district was fitted, as shown in Figure 7a. Water quality in the whole lake was correlated with daily water diversion, and correlations are shown in Figure 7b. From the correlation, it can be seen that water quality increased first and then decreased with increasing water inflow discharge. In general, when the water discharge was about 180,000 m^3^/day, the water quality concentration value was the lowest, and Xuanwu Lake had the best water quality. Water quality data was supplied by the Nanjing Environmental Protection Bureau.

#### 3.1.2. Theoretical Analysis

**Figure 8:**
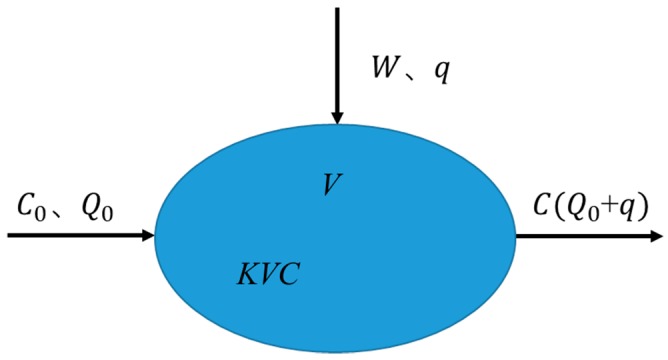
Zero-dimensional model schematic.

A zero-dimensional model was used for water quality analysis [38,39,40]. For a research water body, when the internal water masses are evenly mixed, it can be assumed that the substances flowing into the system are completely dispersed to the entire system, and the entire water body can be regarded as a complete system. The volume V is a complete mixing system. The volume of blowdown is W, the concentration is C, the flow out of the system is $Q_{out}=Q_{0}+q$, and the flow into the system is $Q_{in}=Q_{0}$ [41,42,43], as shown in Figure 8.

$$V\frac{dC}{dt}=W-Q_{out}C+Q_{in}C_{in}-KVC$$

$$K^{\prime }=Q_{out}+KV$$

$$V\frac{dC}{dt}+K^{\prime }C=W+Q_{in}C_{in}$$

$$W+Q_{in}C_{in}\neq 0$$

$$C=\frac{W+Q_{in}C_{in}}{Q_{out}+KV}\left[1-\exp\left(-\frac{Q_{out}+KV}{V}t\right)\right]$$

$${C|}_{t\to \infty }=\frac{W+Q_{in}C_{in}}{Q_{out}+KV}$$

In the formula, W: Pollutant emissions; $Q_{0}$: Flow into rivers and lakes; $C_{0}$: Background concentration of upstream water; K: Water degradation coefficient; V: Water volume.

According to the evaluation result of Xuanwu Lake pollution source, we set the pollutants (excluding water supply) into the lake every day as the same, $W_{TP}$ = 0.3175 kg/day, $W_{TN}$ = 2.1166 kg/day. Because $Q_{0}$, $C_{0}$, C, and V are all known, we solve for the K value:

$$K=\frac{W+Q\left(C_{0}-C\right)}{VC}$$

Then according to the water residence time, $t=\frac{V}{Q}$.

**Figure 9:**
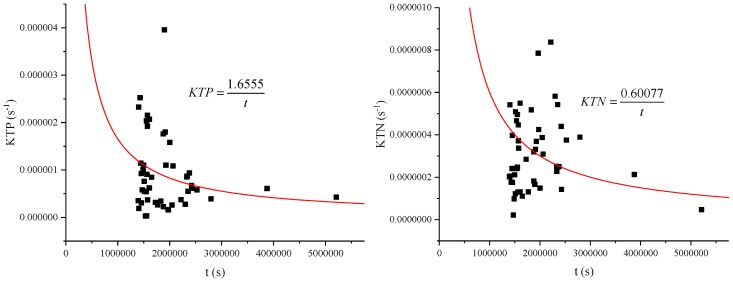
Relationship between water quality degradation coefficient and residence time in Xuanwu Lake. We fit each scatter plot to an inverse function. The equation is: *K* = B1/*t*, *K* is the degradation coefficient, *t* is the residence time, and B1 is constant parameters. The B3 for each curve (left to right) is: 1.6555, 0.60077. The coefficient of determination, R-squared, for each curve (left to right) is: 0.3801, 0.2985.

The relationship between the calculated K and the residence time t was established, and the relationship between the TP degradation coefficient KTP ~ t and the TN degradation coefficient KTN ~ t were fitted, as shown in Figure 9. The degradation coefficient decreased as the residence time increased. The degradation coefficient decreased as the residence time increased, but the product of the two factor was a constant.

Because, we know $K=\frac{W+Q\left(C_{0}-C\right)}{VC}$, it then follows that $KVC=W+Q\left(C_{0}-C\right)$.

Let $KVC=W_{K}$, $W_{K}$ be the amount of degradation, then it follows that $W_{K}=W+Q\left(C_{0}-C\right)$.

As the monitoring shows that the water quality concentration of Xuanwu Lake is greater than the water quality concentration of the inflow, i.e., C > $C_{0}$, let $A=C-C_{0}$, A > 0.

**Figure 10:**
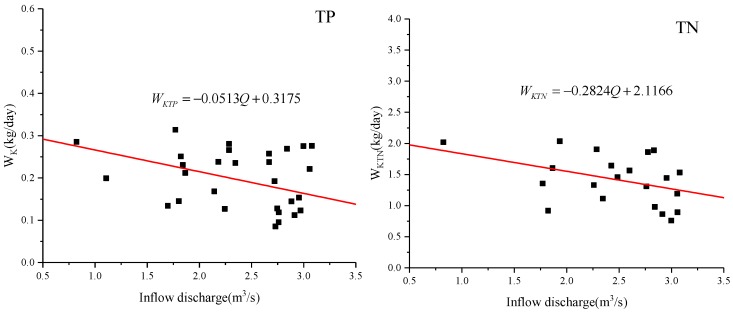
Relationship between amount of degradation and inflow discharge in Xuanwu Lake. We fit each scatter plot to linear function. The equation is: *W* = B1*Q + B2, and B1, B2 are constant parameters. The B1 for each line (left to right) is: −0.0513, −0.2824. The B2 for each line (left to right) is: 0.3175, 2.1166. The coefficient of determination, R-squared, for each line (left to right) is: 0.1872, 0.1795.

$W_{K}=-AQ+W$, degradation was negatively correlated with water diversion, as shown in Figure 10. Although the degradation coefficient decreased with the increase of residence time, the amount of degradation increased with the increase of residence time, that is, the amount of degradation increased with the decrease of water transfer amount.

**Figure 11:**
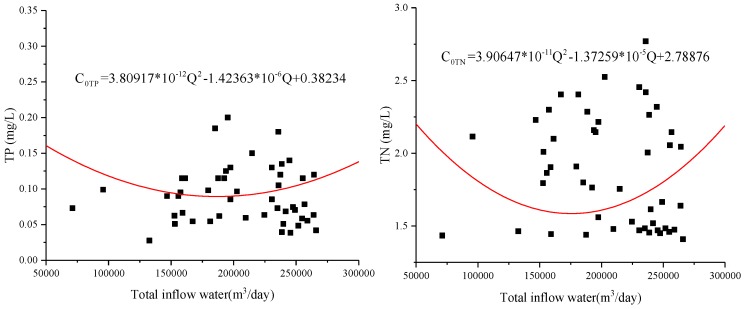
Relationship between diversion water concentration and total inflow We fit each scatter plot to a quadratic function. The equation is: *C* = Intercept + B1*Q + B2*Q^2^, *C* is the water quality concentration, *Q* is the inflow discharge, and Intercept, B1, and B2 are all constant parameters. The Intercept for each curve (left to right) is: 0.3823, 2.7888. The B1 for each curve (left to right) is: −1.4236 × 10^−6^, −1.3726 × 10^−5^. The B2 for each curve (left to right) are: 3.8092 × 10^−12^, 3.9065 × 10^−11^. The coefficient of determination, R-squared, for each curve (left to right) is: 0.3643, 0.2108.

According to the water quality characteristics of Xuanwu Lake in 2014~2017, the concentration of TP was between 0.027 mg/L~0.2 mg/L, and the concentration of TN was between 1.41 mg/L~2.77 mg/L. The relationship between inflow water quality and discharge in Xuanwu Lake is constructed within the water quality concentration interval, as shown in Figure 11.

According to the lake uniform mixing model:

$$C=\frac{W}{Q+KV}+\left(C_{0}-\frac{W}{Q+KV}\right)\exp\left(-\frac{Qt}{V}-Kt\right)$$

According to $t=\frac{V}{Q}$, $KTP=\frac{1.6555}{t}$, $KTN=\frac{0.60077}{t}$, $W_{TP}$ = 0.3175 kg/day, $W_{TN}$ = 2.1166 kg/day

$$C_{TP}=0.0703C_{0TP}+\frac{0.1112}{Q}$$

$$C_{TN}=0.20174C_{0TN}+\frac{1.05549}{Q}$$

*C_0TP_* or *C_0TN_* is the water quality of diversion; *C_TP_* or *C_TN_* is the water quality of Xuanwu Lake.

**Figure 12:**
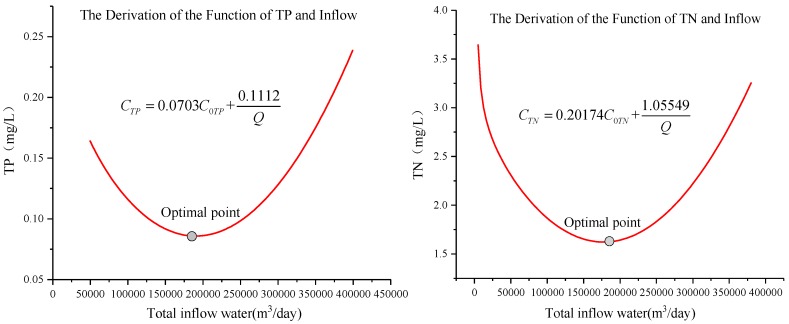
Relationship between diversion water quality and total inflow in Xuanwu Lake. It is clear from the figure that the water quality of Xuanwu Lake first became better and worse after the increase of the flow rate, and the optimal flow rate was 180,000 m^3^/day. At the same time, we can also see from the derivation function that the water quality of Xuanwu Lake has a relationship with the water quality and flow of the diversion water. The water quality of Xuanwu Lake had a positive correlation with the water quality of diversion water, and it had a negative correlation with the inflow discharge of diversion water. When the inflow discharge is less than 180,000 m^3^/day, the volume of water diversion will play a leading role in the water quality of Xuanwu Lake. However, when the water diversion volume is greater than 180,000 m^3^/day, the water quality of diversion will play a dominant role in the water quality of Xuanwu Lake.

The relation between water quality concentration *C* and water quantity *Q* in Xuanwu Lake is shown in Figure 12. According to the theoretical analysis and formula derivation, it can be seen that the water quality of Xuanwu Lake changed for the better and then became worse as the water inflow increased. When the water quality was optimal, the amount was about 180,000 m^3^/day.

### 3.2. Calculation Programs

According to the analysis, the optimal inflow of Xuanwu Lake was 180,000 m^3^/day, so the water quantity of the No.1 water plant was reduced to 50,000 m^3^/day, and the No. 2 water plant was reduced to 130,000 m^3^/day [44,45,46]. Through related research, the minimum flow of water transferred from Xuanwu Lake to urban rivers is 2 m^3^/s, which means that the minimum amount of water supply to Xuanwu Lake is 172,800 m^3^/day. We set the maximum water diversion (3,500,000 m^3^/day), daily water diversion (2,800,000 m^3^/day), and optimal water diversion (1,800,000 m^3^/day) as the calculation plan, taking into account no wind and prevailing winds (southeast wind and northwest wind) [47,48,49]. According to the field survey, the pollutant ratio of the ③ drainage ditch was significant, so the ③ drainage ditch will be blocked during the optimal water diversion [50,51,52], as shown in Table 2.

### 3.3. Calculation Results

#### 3.3.1. Water Diversion without Wind

**Figure 13:**
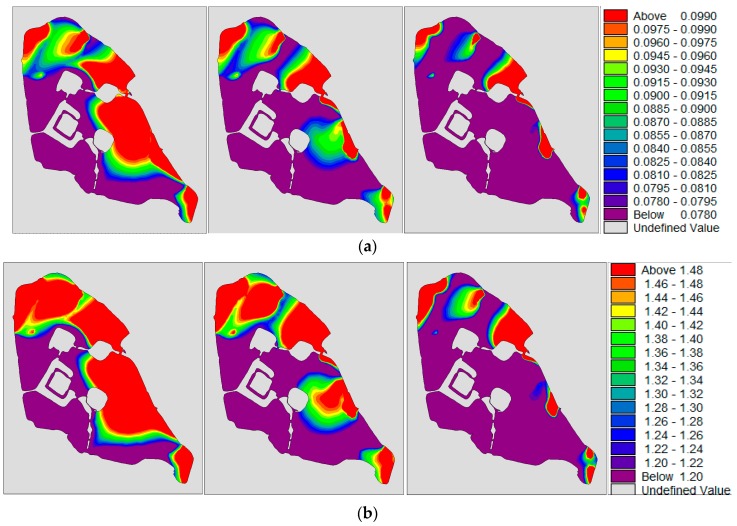

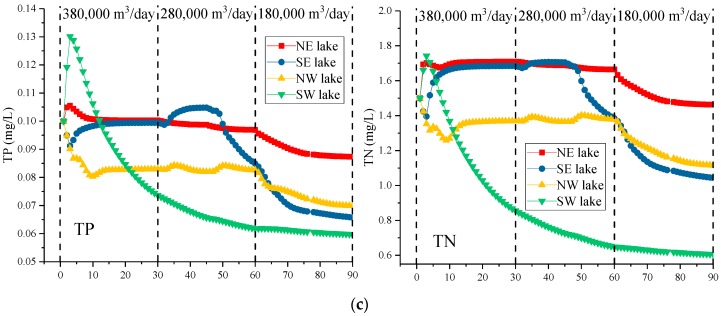
(**a**,**b**) are the concentration distributions of TP (total phosphorus) and TN (total phosphorus) under the no-wind condition. From left to right, the water diversion schemes for 350,000 m^3^/day, 280,000 m^3^/day and 180,000 m^3^/day respectively; (**c**) shows the changes in water quality under different water diversion schemes for different lake districts in Xuanwu Lake.

Depending on the calculation of the two-dimensional mathematical model [4,53,54], the water quality concentration near the sewage outlet was high under the condition of no wind, and the water quality concentrations of the northwest lake and southwest lake are relatively low, as shown in Figure 13.

#### 3.3.2. Water Diversion with Southeast Wind

**Figure 14:**
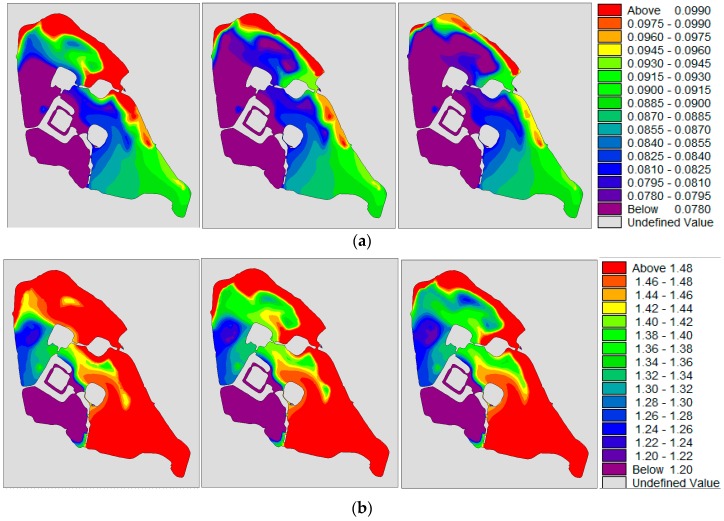

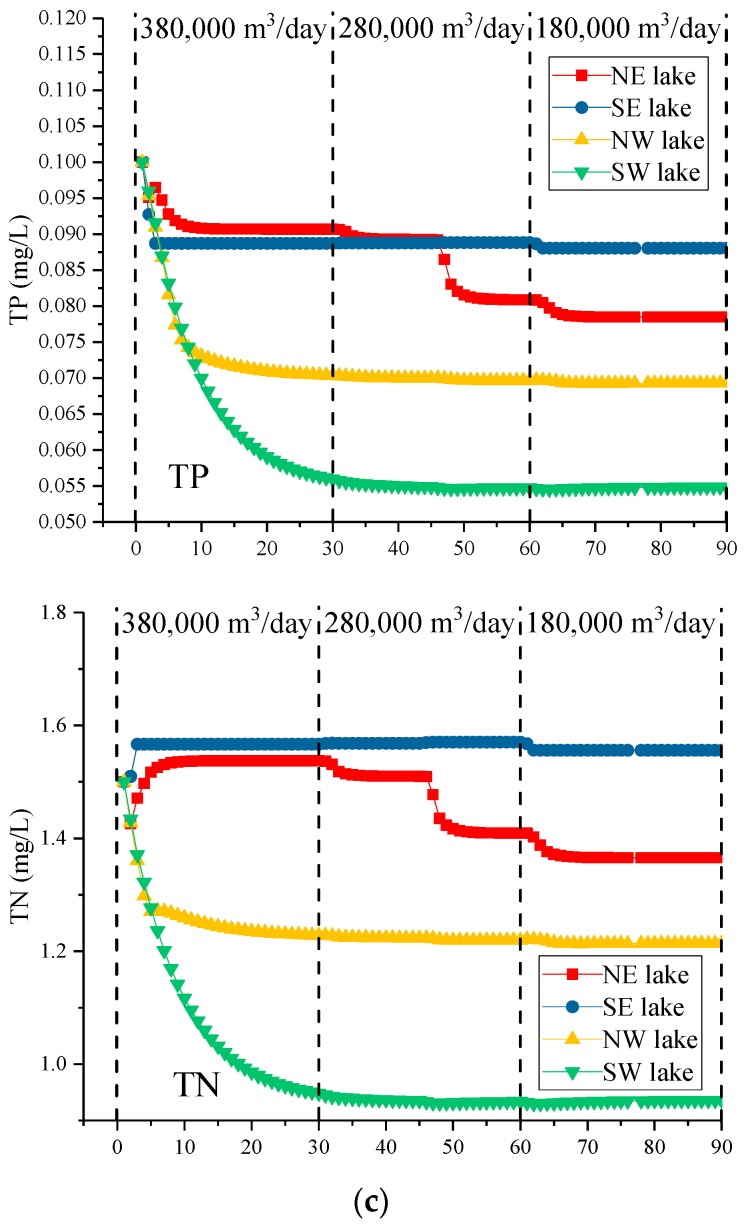
(**a**,**b**) are the concentration distributions of TP(total phosphorus) and TN(total nitrogen) under southeast wind condition. From left to right, the water diversion schemes for 350,000 m^3^/day, 280,000 m^3^/day and 180,000 m^3^/day respectively; (**c**) shows the changes in water quality under different water diversion schemes for different lake districts in Xuanwu Lake.

Under the continuous influence of the southeast wind, when the water quality of Xuanwu Lake was stable, the water quality concentration was SE district > NE district > NW district > SW district, as shown in Figure 14. Under the influence of the southeast wind, the water quality of the lake area was better than that of the no wind condition, and the concentration field distribution was more even than the no wind condition.

#### 3.3.3. Water Diversion with Northwest Wind

**Figure 15:**
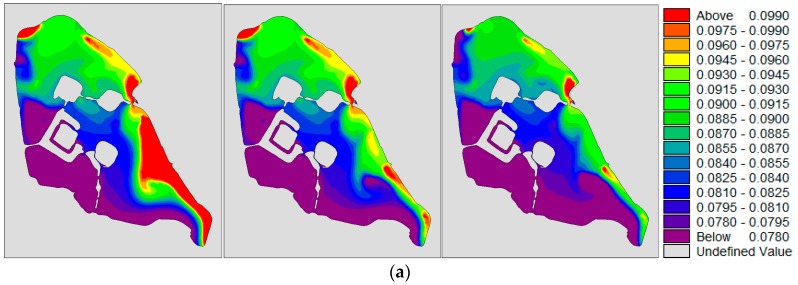

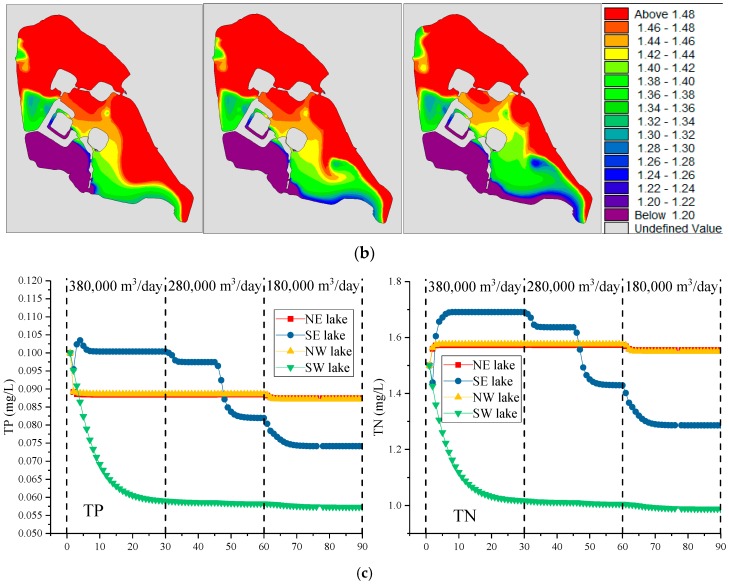
(**a**,**b**) are the concentration distribution of TP(total phosphorus) and TN(total nitrogen) under northwest wind condition. From left to right, water diversion schemes for 350,000 m^3^/day, 280,000 m^3^/day and 180,000 m^3^/day respectively; (**c**) shows the changes in water quality under different water diversion schemes for different lake districts in Xuanwu Lake.

Under the continuous influence of the northwest wind, when the water quality of Xuanwu Lake was stable, the water quality concentration was NW district > NE district > SE district > SW district, as shown in Figure 15. Under the influence of the northwest wind, the water quality of the lake area was better than that of the southeast wind, and the concentration field distribution was more even than the southeast wind condition.

#### 3.3.4. Water Diversion with Different Directions of Wind

**Figure 16:**
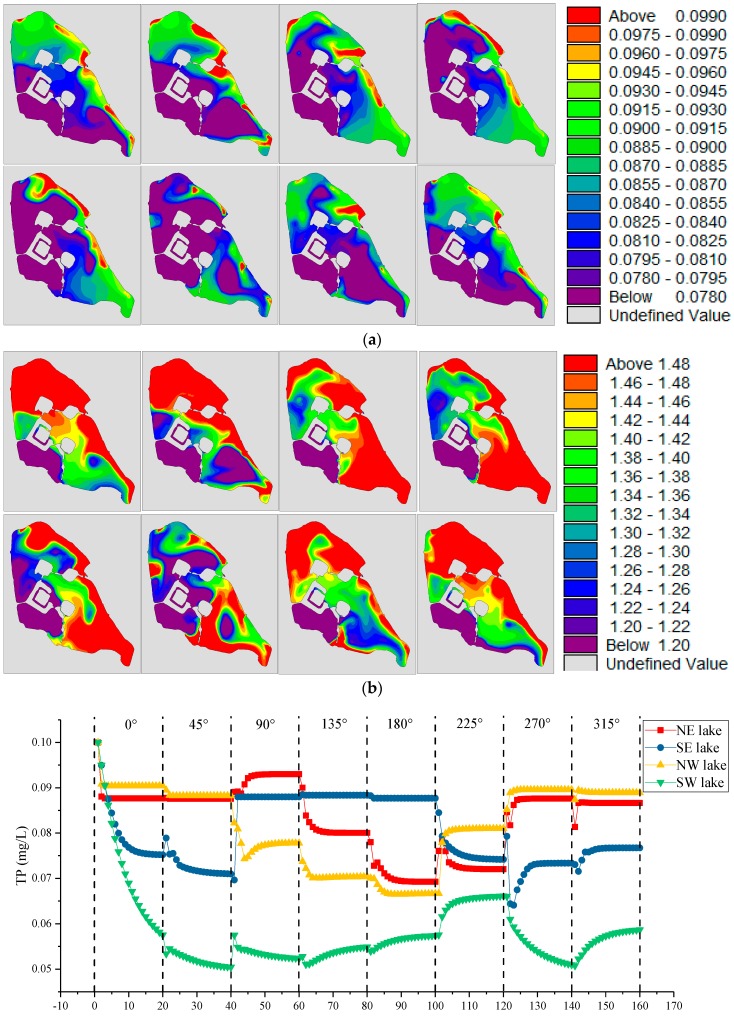

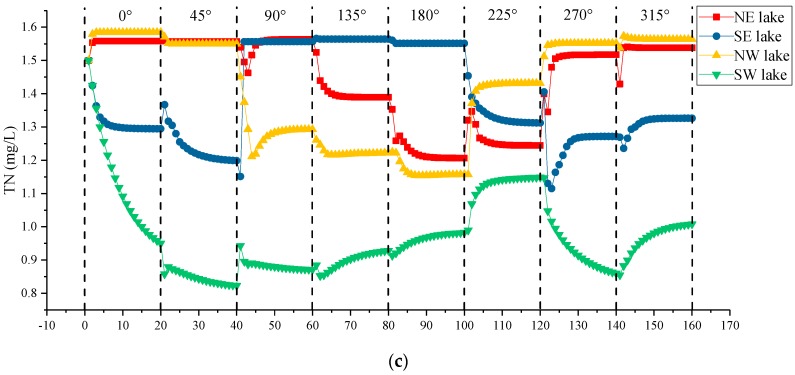
(**a**,**b**) are the concentration distribution of TP(total phosphorus) and TN(total nitrogen) under different directions of wind. From left to right and from the first row to the second row, the wind directions are: Northern (0°), Northeastern (45°), Eastern (90°), Southeastern (135°), Southern (180°), Southwestern (225°), Western (270°), Northwestern (315°); (**c**) shows the changes in water quality under different wind directions for different lake districts in Xuanwu Lake.

Section 3.3.2 and Section 3.3.3 were intended to predict the impact of the prevailing winds on the water quality of Xuanwu Lake. However, human activities have made climate change worse. Section 3.3.4 predicted the impact of the unknown wind direction on Xuanwu Lake. We calculated and compared more different directions of wind with water diversion 180,000 m^3^/day, as shown in Figure 16. Different wind directions result in different water quality concentrations and different concentration distribution fields in different lake districts. The concentration of water in each lake districts varied greatly. In addition to the southwestern wind, the maximum concentration of other districts in other winds was about two-times of the minimum concentration. The relatively close concentration of water quality in the four lake districts was under the influence of the southwest wind. In general, the concentration of water quality in the NE district was the highest, and that in the SW district was the lowest.

#### 3.3.5. Water Diversion with Different Speed of Wind

**Figure 17:**
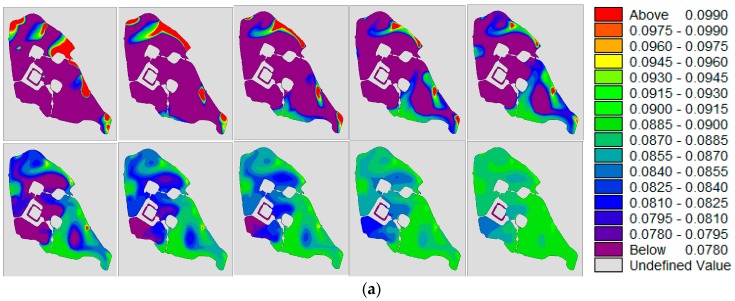

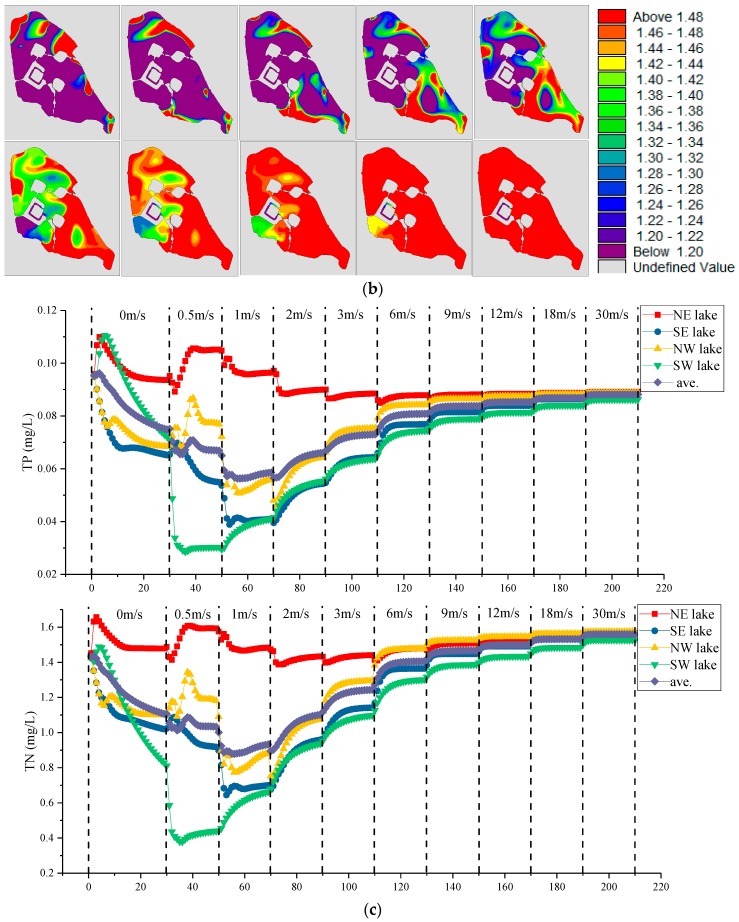
(**a**,**b**) are the concentration distribution of TP(total phosphorus) and TN(total nitrogen) under different speed of wind. From left to right and from the first row to the second row, the wind speeds are: 0 m/s, 0.5 m/s, 1 m/s, 2 m/s, 3 m/s, 6 m/s, 9 m/s, 12 m/s, 18 m/s, 30 m/s; (**c**) shows the changes in water quality under different wind speeds for different lake districts in Xuanwu Lake.

In the case of wind direction (southwestern, 225°), where the water quality of the four lake districts was relatively close, the water quality of the lake in different wind speed was compared, as shown in Figure 17. In general, the concentration of water quality in the NE district was the highest, and that in the SW district was the lowest. Judging from the average water concentration in the four districts, the concentration of water quality in Xuanwu Lake first became smaller and then became larger as the wind speed increased, and eventually became constant. When the wind speed was 1 m/s, the quality of Xuanwu Lake was the best, the total phosphorus concentration is 0.06 mg/L, and the total nitrogen concentration is 0.95 mg/L. The concentration of total phosphorus without wind condition was basically the same as the concentration of water with a wind speed of 3 m/s. The water concentration of total nitrogen under no wind condition was basically the same as the water quality concentration at a wind speed of 2 m/s. When the wind speed was greater than 3 m/s, the wind will deteriorate the lake water quality. Compared to no wind condition, the maximum wind speed will increase the total phosphorus concentration in Xuanwu Lake by 15% and increase the total nitrogen concentration by 40%.

### 3.4. Discussion

The optimal water inflow discharge in this paper is 180,000 m^3^/day, which is quite different from previous [55] research result of 315,000 m^3^/day. In 2007, a large amount of water transfer in Xuanwu Lake could indeed make the water quality better, mainly because the water quality in the lower reaches of the Yangtze River was better [56]. At the same time, when the city’s sewage pipe network lags behind, the amount of sewage flowing into the river is relatively large [57], and the inflow discharge 315,000 m^3^/day [55] can exactly meet the requirements of lake water quality. In the past, urban sewage pipe network was behind and the amount of sewage flowing into the river was relatively large. The amount of water diversion of about 315,000 m^3^/day was just enough to meet the requirements of lake water quality. However, nowadays, a large number of municipal sewage is connected to the sewage treatment plant, but some of the sewage still enters the river, and at the same time the water quality of the lower reaches of the Yangtze River is deteriorating [58]. A large number of water diversion will not make the water quality of Xuanwu Lake better, but will worsen. Lake water is difficult to dilute with high concentration, causing research to shift to the self-degradation of pollutants. In this paper, the water quality of Xuanwu Lake can be achieved by reducing the amount of water transfer and increasing the residence time (15.3 days changed to 23.8 days) of water. When the inflow discharge be less than 180,000 m^3^/day, the water quality of Xuanwu Lake gradually improves as the inflow discharge of water diversion increases. At this time, water quality degradation plays an important role. However, when the inflow discharge exceeds 180,000 m^3^/day, the residence time is short, and the lake becomes a through-water channel, and its self-degradation weakens, and the water quality of diversion plays a major role in the lake water quality. It can be seen from Figure 7, Figure 9, Figure 10 and Figure 11 that the low coefficient of determination does not mean that they are not related, but that they are weakly related and relevant. There is no contradiction between the data and the conclusion. When the data sample is sufficient, it can be concluded that the conclusion is correct. Xuanwu Lake’s sample data is from 2014 to 2017. The number of samples is sufficient. The statistical analysis is reasonable. The conclusion is reliable and consistent with the actual situation.

Relevant studies [59,60,61,62] have shown that the control of the total amount of pollutants in water is crucial for the management of the water environment. For a multiple outlets lake, the outlets are often the river channels of the city. The water quality of the lake will determine the water quality of each river upstream and the total amount of pollutants in the river. The average water quality for multiple monitoring sites in a lake may be up to the standard, but there may be a high water quality concentration monitoring site near an outlet. Water with high concentration of pollutants becomes upstream of a certain city’s river, this kind of water brings about great pollution to the city. The influence of wind direction and wind speed on the concentration distribution of lake pollutants will affect urban river water quality. The more uniform the water quality of the lake is, the more favorable it is to the urban river water quality. For Xuanwu Lake, the water quality concentration is the most uniform under the influence of the SW wind. Relevant studies [63] have shown that the shape of the wind-induced flow field is determined by the wind direction and has little to do with the wind speed. The flow field caused by the SW wind of Xuanwu Lake is conducive to the diffusion of pollutants, reducing the concentration of pollutants in high-concentration outlets, and decreasing the risk of urban river pollution. However, this study shows that wind speed is linked to water quality, which is different from other people’s studies. Under the optimal wind direction, the water quality will become better first with the increase of wind speed, and then it will become worse, and finally it will tend to a fixed value.

## 4. Conclusions

Through the analysis of the measured data of Xuanwu Lake’s inflow discharge and lake water quality, it can be concluded that blindly generous amounts of water diversion cannot make the water quality the best. According to the measured data, theoretical and numerical simulation analysis, the main conclusions are as follows:

(1) From the curve of water quality and daily water inflow discharge in each district was fitted, it can be seen that water quality increased first and then decreased with increasing water inflow discharge in Xuanwu Lake. In general, when the water discharge is about 180,000 m^3^/day, the concentration of pollutants value was the lowest, and water quality of Xuanwu Lake was the best.
(2) For a research water body, when the internal water masses are evenly mixed, it can be assumed that the substances flowing into the system are completely dispersed to the entire system, and the entire water body can be considered as a complete system. The zero-dimensional model was used for Xuanwu Lake water quality analysis. Through theoretical analysis, the relationship between water quality and water diversion was established. We also found that the multiplication of the pollutant degradation coefficient (*K*) and the water residence time (*T*) is a constant (*N*), *K* · *T* = *N*. The water quality changed better first with the increase of inflow discharge, and then became worse, and the optimal water quality inflow discharge is also 180,000 m^3^/day. It shows that the regular analysis of the measured data of Xuanwu Lake is in accordance with the theoretical analysis, which verifies the accuracy of the conclusion.
(3) By constructing a mathematical model of a two-dimensional water environment and setting up various programs, different inflow discharges, wind directions, wind speed, and pollutant emissions are considered. The gradual deterioration of water quality in Xuanwu Lake: ① inflow discharge: 180,000 m^3^/day > 280,000 m^3^/day > 350,000 m^3^/day; ② wind speed: 3 m/s > 0 m/s; ③ wind direction: NW wind > SE wind. Once again, it is proved that under the optimal inflow discharge, proper wind speed and proper wind direction may make the water quality of Xuanwu Lake better.
(4) Through the calculation of eight winds directions at the optimal inflow discharge. Different wind directions result in different water quality concentrations and different concentration distribution fields. The concentration of water in each lake districts varies greatly. In general, the concentration of water quality in the NE district was the highest, and that in the SW district was the lowest. The relatively close concentration of water quality in the four lake districts was under the influence of the SW wind. In addition to the SW wind, the maximum concentration of other districts in other winds was about two-times the minimum concentration. Under the conditions of high-intensity human interference in urban complex and meteorological conditions, the proper wind direction is more uniform distribution of lake pollutants. It is beneficial to control the total amount of pollutants in the river where the outlets are located.
(5) In the SW wind direction, judging from the average water concentration in the four districts, the concentration of pollutants in Xuanwu Lake first became smaller and then became larger as the wind speed increased, and eventually became constant. When the wind speed was 1 m/s, the quality of Xuanwu Lake was the best, the total phosphorus concentration was 0.06 mg/L, and the total nitrogen concentration was 0.95 mg/L. The concentration of total phosphorus without wind condition was basically the same as the concentration of water with a wind speed of 3 m/s. The water concentration of total nitrogen under no wind condition was basically the same as the water quality concentration at a wind speed of 2 m/s. When the wind speed was greater than 3 m/s, the wind will deteriorate the lake water quality. Compared to no wind condition, the maximum wind speed will increase the total phosphorus concentration in Xuanwu Lake by 15% and increase the total nitrogen concentration by 40%. As the wind speed increased, the concentration of pollutants in the lake was more evenly distributed. Under the condition of small wind speed, the scope of pollutant diffusion was small, making the monitoring point water quality not represent the entire lake. It is recommended to conduct water quality monitoring under large wind speed, or to increase monitoring points when the wind speed is small, so that the water quality monitoring results are more realistic.

## Figures and Tables

**Table 1:** Error analysis table.

Time	TP				TN			
	*K* (s^−1^)	Simulation (mg/L)	Measured (mg/L)	Error (%)	*K* (s^−1^)	Simulation (mg/L)	Measured (mg/L)	Error (%)
3 Jan. 2017	1.53 × 10^−6^	0.0811	0.0775	4.65	1.1 × 10^−6^	1.7385	1.7875	2.74
7 Feb. 2017	2.26 × 10^−6^	0.0608	0.0525	15.81	9.26 × 10^−7^	2.1463	2.125	1.00
1 Mar. 2017	1.53 × 10^−6^	0.0793	0.0775	2.39	9.74 × 10^−7^	2.1613	1.9825	9.02
6 Apr. 2017	1.35 × 10^−6^	0.0929	0.0875	6.14	1.27 × 10^−6^	1.5486	1.5525	0.25
4 May 2017	1.01 × 10^−6^	0.1269	0.1175	7.99	1.29 × 10^−6^	1.5589	1.52	2.56
2 Jun. 2017	6.77 × 10^−7^	0.1713	0.175	2.10	1.41 × 10^−6^	1.5040	1.3825	8.79
3 Jul. 2017	1.44 × 10^−6^	0.0988	0.0825	19.70	1.24 × 10^−6^	1.5623	1.5875	1.58
1 Aug. 2017	5.92 × 10^−7^	0.1805	0.2	9.74	1.47 × 10^−6^	1.5150	1.3125	15.43
4 Sep. 2017	6.58 × 10^−7^	0.1980	0.18	10.01	1.13 × 10^−6^	1.7373	1.7475	0.58
2 Oct. 2017	1.21 × 10^−6^	0.1125	0.0975	15.39	8.55 × 10^−7^	2.3060	2.3075	0.07
1 Nov. 2017	8.61 × 10^−7^	0.1141	0.1375	17.05	1.1 × 10^−6^	1.9879	1.7325	14.74
4 Dec. 2017	1.25 × 10^−6^	0.1113	0.095	17.20	1.1 × 10^−6^	1.9409	1.7575	10.44

**Table 2:** Calculation Programs.

Wind Direction	Diversion Water (m^3^/day)	③ Drainage Ditch	Wind Speed (km/h)
No wind	350,000	Open	-
	280,000	Open	-
	180,000	Close	-
Southeast (135°)	350,000	Open	8
	280,000	Open	8
	180,000	Close	8
Northwest (315°)	350,000	Open	8
	280,000	Open	8
	180,000	Close	8
